# Exploring the Influence of Determinants on Behavior Intention to Use of Multiple Media Kiosks Through Technology Readiness and Acceptance Model

**DOI:** 10.3389/fpsyg.2022.852394

**Published:** 2022-03-31

**Authors:** Michael Yao-Ping Peng, Xin Yan

**Affiliations:** ^1^Business School, Foshan University, Foshan, China; ^2^College of Business, City University of Hong Kong, Kowloon, Hong Kong SAR, China

**Keywords:** technology readiness, technology acceptance model, interactivity, behavior intention to use, multiple media kiosks

## Abstract

The use of multiple media kiosks (MMKs) is witnessing an increasingly strong uptrend in 24-h chain convenient stores in developed countries. However, as the functions of MMKs intensifies and increases, how to retain consumers has been a topic that raises concerns of managers and researchers. In this study, we adopt the integrated technology acceptance model, which combines technology readiness and acceptance model with interactivity that serves as the moderating factor, with the purpose of discussing the relationships among all these variables and their impacts on behavior intention to use. Through the cross-sectional survey and purposive sampling, a total of 623 copies of questionnaire from Taiwan were collected in this study. Smart-PLS for PLS-SEM was applied in the structural model to conduct a verification of the hypotheses and comparative analysis in this study. The results show that all hypotheses were significantly supported; technology readiness has a positive impact on perceived ease to use and perceived usefulness; and interactivity also positively moderates the relationship among perceived ease to use, perceived usefulness and attitude toward using. Our results will offer more insights and advice concerning theories of service technology.

## Introduction

The 24-h chain convenient stores have become an important channel for buying daily necessities in Asia, as their density of distribution increases (Chang et al., 2016; Kwak and Cho, 2019; Hsiao et al., 2020). In order to improve consumer experience and functionality of convenient stores, firms launched multiple media kiosks (MMKs) to expand their service scope (Brengman et al., 2021; Lao et al., 2021). MMK is a self-server terminal that is commonly seen in the retail channel. Its functions may vary from national cultures or channels (Lee, 2015; Kwak and Cho, 2019; Hsiao et al., 2020), but it is generally provided with a touch screen, supplemented with multi-media interface and voice effect, to guide consumers to finish the consumption process quickly (Chang et al., 2016; Chang and Chen, 2021). Self-service technology, which is self-server terminal, is simply a kind of intelligent human-computer interaction equipment, in which the user operates and completes the business through a device according to the provided instructions. This kind of self-server terminal not only makes extremely high profits for a retail store in the least space, but also allows consumers to have a delightful and novelty experience (Ku and Chen, 2012; Grewal et al., 2020; Brengman et al., 2021; Lao et al., 2021), thus creating new added value for retailers (Kwak and Cho, 2019). Although it is an interesting experience platform, firms are not suggested to focus only on MMKs, because MMKs increase the learning burden for consumers, especially the aged or children (Chang et al., 2016; Hsiao et al., 2020). A large number of previous studies have discussed the impact of new technologies on users’ intention to use by the Technology Acceptance Model (TAM) (Min et al., 2019; Rafique et al., 2020), but there are few studies that discuss psychological factors caused by new technologies adopted in convenience stores (Kim et al., 2017). Thus, this study explores the consumer acceptance of MMKs in convenient stores using the TAM in a updating framework, involving interactivity as moderator.

Based on the above, the contribution of the present study is threefold. First, this study empirically investigates consumer perception and attitude toward MMKs in convenience stores. Most of previous studies focused on innovative services in such industries as banking, information service and education service, and verified the positive perception of users against new technology media. But seldom of them have had further discussions on MMKs that are subject to continuous updating of system contents. Second, this study verifies users’ acceptance of MMKs based on the TRASM framework, and uses TR as the antecedent to discuss its impact on the original TAM. Third, interactivity, which is important but often ignored, is taken as the moderator in this study to explain to what extent users are involved in innovative services and how to strengthen the use and cognition of new technologies.

### Research Approach

Over the past three decades, scholars have made a considerable number of studies using the TAM. In addition to improvements for the original TAM, these studies also introduced other theories in their discussions, especially those about marketing, education and management (Wallace and Sheetz, 2014; Mortenson and Vidgen, 2016; Rafique et al., 2020; Wang et al., 2020). In despite of these previous studies, TAM is still one of the most suitable theoretical models for exploring users’ responses to new technologies (Wallace and Sheetz, 2014; Mortenson and Vidgen, 2016; Kim et al., 2017; Humbani and Wiese, 2019). However, for MMKs subject to fast-updating technologies, the mere TAM is not enough to explain technology adoption behaviors of consumers (Kim et al., 2017; Rafique et al., 2020). In order to enrich understandings and insights, it is necessary to build a complete interpretation model to discuss the psychological process of individuals perceiving the technology value (Wallace and Sheetz, 2014; Manis and Choi, 2019; Rafique et al., 2020; Wang et al., 2020; Lao et al., 2021). Some scholars indicated that individual differences serve as an important factor in making TAM more generalized (Manis and Choi, 2019) and explaining how the technology value perception of individuals affect their technology adoption behaviors or intentions (Wallace and Sheetz, 2014; Lin and Kim, 2016; Min et al., 2019; Wang et al., 2020). As a result, we integrated technology readiness (TR) with traditional TAM as an antecedent that affects TAM, with the purpose of discussing consumers’ intention to use MMKs in convenience stores (Parasuraman and Colby, 2015). Different from TAM that is based on user motivation, the TAM integrating TR can reflect users’ positive (i.e., optimism and innovativeness) and negative (i.e., discomfort and insecurity) mental readiness against innovative technologies. TR can be regarded as a tendency to or a belief in achieving different types of goals through consumers “individuals” acceptance and use of new technologies (Jin, 2013; Oh et al., 2014; Kim and Chiu, 2019; Chiu and Cho, 2020; Chang and Chen, 2021), indicating that consumers with a higher level of TR tend more to accept and use new technologies and functions to solve problems in life (Parasuraman and Colby, 2015; Chang and Chen, 2021). Combined with the Technology Readiness and Acceptance Model (TRAM) proposed by Lin et al. (2007), this study discusses the mental process of consumers when they use technologies equipped for MMKs in convenience stores.

In recent years, scholars have pointed out that the scope of studies should not be limited to individuals’ unilateral cognition and attitudes toward new technology media, but should be expanded to the interactivity between them (Lin and Kim, 2016; Fan et al., 2017; Manis and Choi, 2019). They emphasized that the technology adoption of consumers is a process view (Kallweit et al., 2014; Roy et al., 2018; Kim and Lee, 2019; Pillai et al., 2020), but not a result orientation (Kim et al., 2017; Humbani and Wiese, 2019; Rafique et al., 2020; Wang et al., 2020). In order to present a more complete conceptual framework, this study proposes that consumers with a higher interactivity may be more willing to adopt/use MMKs. Interactivity can be regarded as an amicable using experience, which means that new technology media allow users to access and involve contents of new technologies more easily (Barreda et al., 2016; Janlert and Stolterman, 2017; Kim and Lee, 2019). A majority of previous studies used TAM to discuss the positive impact of perceived ease of use and perceived usefulness on users’ intention to use (Lin and Kim, 2016), but ignored the important moderating role of interactivity between innovative technologies and users (Schultz, 2017). Interactivity not only helps increase user stickiness to innovative technologies and services and improve the habit of using them (Fan et al., 2017; Kim and Lee, 2019), but also enriches the theoretical development of TRAM. Therefore, this study uses interactivity as a moderator to discuss its role in changing relationships among perceived ease of use, perceived usefulness and use intention.

## Literature Review and Hypotheses Development

### Technology Acceptance Model

Research subjects about how individuals think, perceive and behave are commonly seen in social sciences (Lin and Kim, 2016). And TAM was often applied to discuss the theoretical models of individuals’ perceived attitude toward acceptance and use of technology media (Davis, 1989; Mortenson and Vidgen, 2016; Kim et al., 2017). TAM is a theoretical model developed for the impact of individuals’ perception and emotion on the use of technology media based on the theory of reasoned action (TRA) (Fishbein and Ajzen, 1975; Rauniar et al., 2014; Wallace and Sheetz, 2014; Manis and Choi, 2019).

In previous studies, TAM was used to probe into consumer attitude toward the use of innovative technologies (Ho et al., 2013; Kim et al., 2017; Manis and Choi, 2019), and to explain consumer perception and cognitive attitude toward contents in the virtual network (Teo, 2009; Lin and Kim, 2016; Rafique et al., 2020; Wang et al., 2020). In potential dimensions of TAM, individuals’ attitude toward using will affect their behavioral intention to use; the perceived usefulness of technology media will affect individuals’ attitude toward using; the perceived ease of use of technology media will affect individuals’ the attitude toward using; the perceived usefulness will affect individuals’ behavioral intention to use; the perceived ease of use will affect individuals’ perceived usefulness (Manis and Choi, 2019; Rafique et al., 2020). External variables refer to other factors that may affect individuals’ perceived usefulness and perceived ease of use toward technology media (Wallace and Sheetz, 2014; Lin and Kim, 2016; Mortenson and Vidgen, 2016; Humbani and Wiese, 2019). TAM-based theoretical models continue to increase, and different conclusions can be inferred due to differences in objects, technology media and academic sectors (Mortenson and Vidgen, 2016; Rafique et al., 2020; Wang et al., 2020). However, after years of research, the focus is still put on the relationships among perceived usefulness, perceived ease of use, attitude toward using, and behavioral intention to use (Rauniar et al., 2014; Wallace and Sheetz, 2014; Lin and Kim, 2016).

For general user context such as the adoption of MMKs, the use intention of respondents cannot be sufficiently explained with few variables only. There are few studies using TAM to further discuss multiple social and behavioral factors related to the specific utilization of MMKs in convenience stores among consumers. In developed countries, MMKs in convenience stores provide consumers with diversified services to solve extensive problems encountered in daily life such as tax payment, mobility ticket buying, and tuition payment (Grewal et al., 2020). Convenience stores often update contents and interface of MMKs for market extension. However, the difficulty in operation also increases while more innovative services are introduced. With the purpose of investigating users’ intention to use MMKs, scholars have also put forward that TAM factors alone may be insufficient to predict acceptance of technology, and have suggested to include other variables (Edmunds et al., 2012; Lin and Kim, 2016). In this study, TR and interactivity are added to extend the original TAM model, and a verifiable conceptual model is built to understand relationships between variables.

Most of previous studies based on the TAM framework have proposed consistent hypotheses (Rauniar et al., 2014; Min et al., 2019; Wang et al., 2020) to understand the role of individual factors played in the use of innovative technologies. And the core variables include perceived usefulness, perceived ease of use, attitude toward using and behavioral intention to use (Rauniar et al., 2014; Lin and Kim, 2016; Min et al., 2019). Scholars have defined attitude toward using as the overall emotional responses generated while individuals use innovative technologies and media. The behavioral intention to use was defined as the extent to which individuals are willing to continue to use new technologies and media (Rauniar et al., 2014; Lin and Kim, 2016). If consumers/users form an emotional bond of fondness when using MMKs, they will have a stickiness in terms of use context (Manis and Choi, 2019; Brengman et al., 2021), subconsciously generating a belief that MMKs will bring with usage advantage (Lao et al., 2021). As argued by scholars on attitude loyalty and behavior loyalty, a good emotion and attitude need to be cultivated before generating any positive behavioral intention to use (Rauniar et al., 2014; Min et al., 2019; Wang et al., 2020). Thus, this study makes a hypothesis as follows:

H1: Attitude toward using has a positive impact on behavioral intention to use.

As scholars pointed out, the two most influential factors in TAM are perceived usefulness and perceived ease of use (Wallace and Sheetz, 2014; Lin and Kim, 2016; Rafique et al., 2020; Wang et al., 2020), which have a strong impact on individuals’ attitude toward using and behavioral intention to use innovative technologies and media (Rauniar et al., 2014; Manis and Choi, 2019; Min et al., 2019). Perceived usefulness was defined as the extent to which individuals believe that the use of innovative technologies and media can enhance their action efficiency; perceived ease of use was defined as the extent to which individuals believe that innovative technologies and media can be used freely (Min et al., 2019; Rafique et al., 2020). In particular, if individuals recognize that no additional effort or learning cost is required in the process of using innovative technologies and media, their perceived benefits and perceived functionalities will enhance (Rauniar et al., 2014; Manis and Choi, 2019; Wang et al., 2020), and they will also develop a preference and favorable attitude toward such use (Lao et al., 2021). In other words, consumers will have a positive attitude toward using if they perceive that the convenience of MMKs will bring them with a high level of functional benefits. Therefore, the researchers postulate the following hypotheses:

H2: Perceived ease of use has a positive impact on perceived usefulness.

H3: Perceived ease of use has a positive impact on attitude toward using.

Perceived usefulness is an intuitive feeling of consumers when they find the efficiency advantages brought by the use of innovative technologies and media. It will gradually become a positive experience cognition (Min et al., 2019) with the accumulation of use experience (Rauniar et al., 2014; Wallace and Sheetz, 2014), which further decides future attitude toward using and behavioral intention to use (Manis and Choi, 2019; Rafique et al., 2020). As argued by scholars, perceived usefulness is an important factor that plays the role of psycho-cognitive shift in consumers’ decision-making process, as well as a cause that stimulates consumers’ act of consequence (Rauniar et al., 2014; Lin and Kim, 2016; Rafique et al., 2020). Therefore, hypotheses are made as follows:

H4: Perceived usefulness has a positive impact on attitude toward using.

H5: Perceived usefulness has a positive impact on behavioral intention to use.

### Technology Readiness

TR is a tendency for people to accept and use new technologies to help them achieve family or work goals (Parasuraman, 2000; Parasuraman and Colby, 2015). The differences between TR and TAM lie in that the former believes that people may also have anxieties or sense of insecurity in face of new technologies, in addition to positive feelings. Parasuraman (2000) conducted a group interview through cooperating with Rockbridge Associates in order to understand the positive and negative perceptions of its customers on technologies. Four variables of TR were proposed based the research findings, namely optimism, innovativeness, discomfort, and insecurity. Specifically, optimism and innovativeness are TR’s enablers, while discomfort and insecurity are inhibitors (Jin, 2013; Oh et al., 2014; Kim and Chiu, 2019; Chiu and Cho, 2020). Marketing personnel evaluate the degree of application of new technologies in customers’ interaction with the firm, the types of technologies introduced, the speed of implementation, and the customer support required (Chiu and Cho, 2020). TR is considered as an individual difference variable, reflecting people’s general attitude toward new technologies (Blut and Wang, 2020).

In decision-oriented studies, TR is often used as a psychological variable, especially under the circumstance that technology-based innovation is the key (Chiu and Cho, 2020). As shown in some studies, TR is correlated with individuals’ higher adoption rate of technology-mediated services in family and work, including online banking service, mobile technology, social robot, self-checkout terminal, remote service, online taxation system and cloud computing (Blut and Wang, 2020). Parasuraman and Colby (2015) used TR index on 127 researchers in 30 countries. They found that although TR has been widely applied by marketing scholars, there is still a conflict and confusion over its dimensions: complex multiple dimensions vs. simple single dimension, resulting in inconsistent and incompatible conclusions. In order to draw a more accurate picture of consumer behaviors, TR can be a consideration when discussing the development of self-server technology (SST). As a result, many subsequent studies were conducted to figure out relationships among TR, satisfaction for SSTs and behavioral intention to use. And all these studies showed that TR has a significant impact on SSTs (Lin and Hsieh, 2007). Furthermore, TR focuses on the measurement of users’ cognition on new technologies, because TR is used to measure users’ tendency in believing that new technologies can help them complete goals, but not to explain users’ behavioral pattern. Thus, Lin et al. (2007) developed a model combining TR with TAM—TRAM (Technology Readiness into Technology Acceptance Model).

As scholars indicated, TR has a significant impact on technology usage (Jin, 2013). Innovative technologies and media may cause a confusion as to their usage. This will further lower users’ willingness to use them and indirectly increase the difficulty for marketing personnel in making decisions on marketing activities (Giebelhausen et al., 2014; Van Doorn et al., 2017). In this case, it is more important for marketing personnel to understand consumers’ readiness to adopt technologies (Blut and Wang, 2020). However, scholars are at odds over relationships between TR and technology usage. Some scholars argued that TR has a significant impact on technology usage (Rahman et al., 2017), while others believed that there is no correlation between them (Chen et al., 2009). One of the possible reasons is that there are important mediators between them, as same as the perceived usefulness and perceived ease of use that are integrated in TAM (Lin et al., 2007; Oh et al., 2014; Parasuraman and Colby, 2015). Moreover, many studies have also demonstrated that TR has a significantly positive impact on perceived usefulness and perceived ease of use (Oh et al., 2014; Chen and Lin, 2018; Kim and Chiu, 2019; Chiu and Cho, 2020). With the purpose of understanding the impact of consumers’ technology readiness to use smart devices in multiple retailing channels on usage of MMKs in convenience stores, hypotheses are postulated as follows:

H6: Technology readiness has a positive impact on perceived usefulness.

H7: Technology readiness has a positive impact on perceived ease of use.

### Interactivity

Interactivity is a quite important concept in the marketing domain (Lee, 2005; Fan et al., 2017). Despite the absence of a very suitable scope or definition (Janlert and Stolterman, 2017), many scholars are still trying to find a suitable dimension for it. This study uses the three dimensions defined by when they discussed the impact of perceived interactivity on e-loyalty, i.e., user control, connectedness and responsiveness. Specifically, user control refers to users’ ability to control information contents and display; connectedness refers to whether customers share their experience of using products or services with other users; responsiveness refers to the ability to response to customer expectation (Lee, 2005; Janlert and Stolterman, 2017).

Interactive technology is defined as methods, tools or devices, through which different entities (individuals, machines or organizations) accelerate or facilitate consumption transactions between them (Varadarajan et al., 2010). The word “interactive” also represents the following characteristics of corresponding communications media: bidirectionality, timeliness, mutual controllability and responsiveness (Bolton and Saxena-Iyer, 2009). The evolution of interactive technology also has a positive impact on consumer experience, in addition to reflecting the development of information technology (Kim and Forsythe, 2008). Besides, some literature stated that interactive technology in the form of the Internet can help retailers manage customer relations (Shah and Murtaza, 2005; Barreda et al., 2016). All these conclusions show that customers’ involvement in consumption can be enhanced if retailers adopt appropriate interactive technologies (Fan et al., 2017). The application of mobile marketing in multi-channel strategies and multi-media context can be considered as a promotion of products through mobile devices, channels or technologies (Shankar and Balasubramanian, 2009; Kim and Lee, 2019). With the bandwidth expansion and the introduction of more advanced mobile technologies, mobile marketing is witnessing a growing impact (Shankar and Yadav, 2010; Schultz, 2017), and managers are also taking more control in the consumer decision-making process by virtue of mature mobile technologies. Both interactive technologies and mobile marketing contain one key driver—Internet. In TAM, the Internet is regarded as an element that is contributable to the enhancement of perceived usefulness (Barreda et al., 2016; Fan et al., 2017). For technologies such as location-based services provided via mobile applications, the Internet can also enhance the consumer utility, facilitating customers to use such applications in consumption (Shankar et al., 2010; Kim and Lee, 2019).

A lot of literature discussed the impact of interactivity on use experience of online shopping or social network sites (Zhao and Lu, 2012; Kim and Lee, 2019), and suggested that web page designers should increase page interactivity to attract and retain customers while emphasizing web page security (Schultz, 2017). Knowing that interactivity is helpful for the internet service design, Shina et al. (2012) argued that consumers’ perception over interactivity plays a significant moderating role in the relationships between the perceived usefulness and perceived ease of use and the attitude toward using. Similar research findings were also suitable for the Web Acceptance Model (WAM) proposed by Castañeda et al. (2007). Thus, this study makes a hypothesis that the higher the interactivity that MMKs provides, the stronger the impact of the perceived usefulness and perceived ease of use on the attitude toward using. Further, hypotheses are developed as follows:

H8: Interactivity positively and significantly moderates the relationship between perceived ease of use and attitude toward using.

H9: Interactivity positively and significantly moderates the relationship between perceived usefulness and attitude toward using.

**FIGURE 1:**
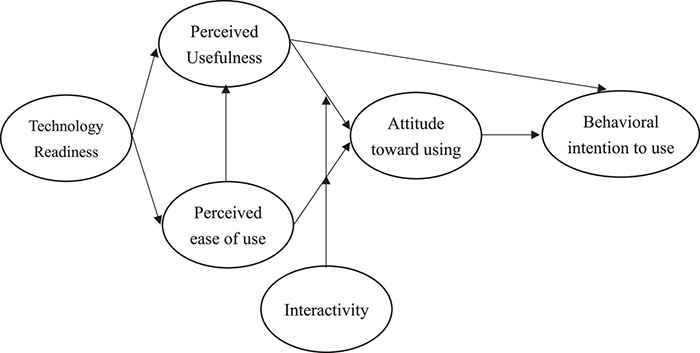
Research framework.

Given the above hypotheses, this study puts forward the research framework (Figure 1) as follow:

## Methodology

### Research Approach

The research objects of this study are mainly consumers who have ever used MMKs in convenience stores and have the ability of consuming on mobile devices and the experience in shopping online. Since Taiwan has the most densely distributed convenience stores in the world, and computers and smart phones have been widely popularized, no considerable differences among sampling objects will be resulted from factors such as place of residence or education background. This study adopted the questionnaire survey approach. The questionnaire survey is a popular and extensively used research technique for quick collection from the target population. Before the formal test, items were translated bidirectionally and then examined to ensure the comprehensiveness and adaptability of questionnaire items and face validity. In addition, a pilot test was conducted using a small size of samples (50 questionnaires) to amend items with unclear semantics or that may cause misunderstanding.

Convenience stores in Taiwan have high popularity and density. According to the news from Taiwan’s “Fair Trade Commission” released a survey on July 20, 2021, that, on average, Taiwan people visit supermarkets 137 times a year, consuming NT $84.16 each time, and the data continues to rise. In terms of stores, by the end of 2020, the main chain convenience stores in Taiwan include 7–11, FamilyMart, Hi-Life, OKmart and TSC Million, with a total of 1,198,500 stores; On average, there is one chain convenience store for every 1966 people, and the density of convenience stores has increased year by year. Based on above reasons, we consider our sampling design seriously with standards. The purposive sampling was adopted in this study. The object population of this research are mainly Taiwan consumers who have used MMKs in convenience stores, and have the ability of consuming on mobile devices and the experience in shopping online, with standards of brands (7–11 and Family Mart), frequency of use (more than twice a month). Questionnaires were issued and collected during the period from July 2020 to September 2020, and a total of 600 valid questionnaires were collected. In the questionnaire, participants were informed of the research purpose, research ethics and low risks, and the questionnaire information was processed in an anonymous way. With the intention of reducing errors caused in the process of inferring and estimating the sample populations through samples, and enhancing the representativeness of research samples, this study also established filling standards during sampling, including brands, frequency of use and targeted age group of convenience stores. The brands of convenience stores are limited to 7–11 and Family Mart. As for the frequency of use, respondents were asked the number of times of visiting convenience stores within 1 month, and those who visit convenience stores for less than twice are deleted. The age group is limited to the range from 20 to 70 years old.

In addition, a Harman’s single-factor test was employed in which all variables were subjected to a principal component analysis (PCA) with varimax rotation. Ten components with eigenvalues greater than 1.0 were obtained. The largest component accounted for 32.4% of the variance, which suggests that the data do not suffer from common method bias. In terms of the sample composition, the respondents consist of male and female about by half; the age groups were divided by every 10 years, and every age group from 20 years old accounted for about 10%, except that there were few respondents from the age groups above 60 years old; a majority of respondents graduated from college (45.3%); about 78.3% of respondents have a disposable income less than USD 1,000. Respondents who use MMKs for three to five times every month accounted for 79.6 and 63.3% of respondents would use MMKs for 3–5 min every time.

This study tested the hypotheses via structural equation modeling (SEM). In order to verify the validity and reliability, confirmatory factor analysis (CFA) was performed adopting IBM-AMOS 23.0. Finally, partial least squares structural equation modeling (PLS-SEM) was used to verify the structural model via Smart-PLS 3.0.

### Measures

Measure variables in the original TAM include perceived usefulness (PU), perceived ease of use (PEU), attitude toward using (AU), and behavioral intention to use (BIU). The scale used in this study was designed by reference to the research scale developed by Davis (1989), so every measure variable has three items. As for the technology readiness, we incorporated Parasuraman and Colby’s (2015) TRI; this uses 12 items (four per construct) to measure customers’ levels of optimism, innovativeness, discomfort and insecurity (Parasuraman and Colby, 2015). As defined in previous research, interactivity refers to the status that consumers can obtain information required from technology media and generate responses and connected activities (Cyr et al., 2009). Based on the research of Lee (2005), this study takes user control, connectedness and responsiveness as measure variables of interactivity. This study uses 9 items to measure customers’ levels of user control, connectedness and responsiveness. All scales use self-reported measures based on 7-point Likert-type response formats, from 1 (“completely disagree”) to 7 (“completely agree”).

## Analysis and Results

### Assessing Measurement Model

Table 1 shows the results: Cronbach’s α scores are from 0.845 to 0.913, showing the high internal consistency of all constructs. Similarly, the combined reliabilities of all constructs are high, ranging from 0.920 to 0.940. Moreover, we measured convergent validity and discriminant validity. The CRs of all constructs are above 0.7, and the AVEs are higher than 0.50, showing sufficient convergent validity. Furthermore, to examine discriminant validity, we compared the square root of the AVE and the cross-correlations among the latent constructs. The square root of AVE for each latent construct (see Table 1) is greater than its cross-correlation with other constructs, confirming discriminant validity.

**TABLE 1:** Measurement properties.

	1	2	3	4	5	6	7	8	9	10	11
1 PU	*0.905*										
2 PEU	0.787	*0.885*									
3 AU	0.715	0.702	*0.875*								
4 BIU	0.663	0.643	0.660	*0.891*							
5 Optimism	0.501	0.527	0.495	0.584	*0.858*						
6 Innovativeness	0.652	0.655	0.593	0.638	0.637	*0.883*					
7 Discomfort	0.537	0.509	0.552	0.536	0.539	0.602	*0.870*				
8 Insecurity	0.578	0.553	0.602	0.571	0.577	0.616	0.714	*0.930*			
9 User control	0.532	0.498	0.552	0.540	0.540	0.563	0.662	0.790	*0.880*		
10 Connectedness	0.560	0.556	0.571	0.591	0.533	0.612	0.594	0.647	0.665	*0.897*	
11 Responsiveness	0.588	0.556	0.539	0.553	0.512	0.598	0.561	0.564	0.567	0.627	*0.960*
Mean	5.05	5.06	4.88	5.03	4.82	4.18	4.29	4.13	4.86	5.13	5.12
SD	1.15	1.17	1.11	1.13	1.29	1.41	1.48	1.65	1.29	1.20	1.28
Cronbach’s α	0.883	0.845	0.836	0.873	0.841	0.855	0.844	0.913	0.873	0.851	0.864
AVE	0.819	0.783	0.766	0.793	0.736	0.780	0.757	0.865	0.775	0.804	0.921
CR	0.931	0.915	0.908	0.920	0.893	0.914	0.903	0.950	0.911	0.925	0.933

*Italicized values mean squared root of AVE values.*

### Inner Model Analysis

Partial least squares structural equation modeling (PLS-SEM) was adopted to construct the structural model; specifically, verification of the structural model was performed using SmartPLS 3.0 (path analysis). According to this study assessed the *R*^2^, beta (β) and *t*-value. Their suggestions also emphasized the predictive relevance (*Q*^2^) as well as the effect sizes (*f*^2^). In the structural model, *R*^2^ values obtained for perceived usefulness (*R*^2^ = 0.431), perceived ease of use (*R*^2^ = 0.356), attitude toward using (*R*^2^ = 0.459) and behavioral intention to use (*R*^2^ = 0.523) were larger than 0.3. Prior to hypotheses testing, the values of the variance inflation factor (VIF) were determined. The VIF values were less than 5, ranging from 1.183 to 2.883. Thus, there were no multicollinearity problems among the predictor latent variables.

**FIGURE 2:**
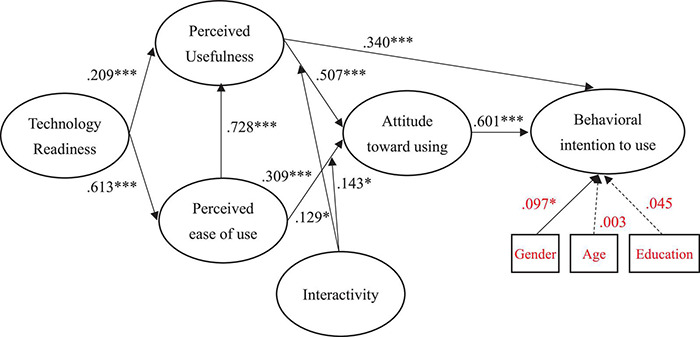
Path Coefficients of Structural Model. * if *p* < 0.05; ^***^ if *p* < 0.001.

Figure 2 and Table 2 shows the results of the hypothesized relationships and standardized coefficients in inner model. The results showed that attitude toward using (β = 0.601, *f*^2^ = 0.136, *p* < 0.001) was significantly related to behavioral intention to use, which supporting H1. Moreover, perceived ease of use was positively and significantly related to perceived usefulness (β = 0.728, *f*^2^ = 0.366, *p* < 0.001) and attitude toward using (β = 0.309, *f*^2^ = 0.182, *p* < 0.001), supporting H2 and H3. In addition, our results found that perceived usefulness was positively and significantly related to attitude toward using (β = 0.507, *f*^2^ = 0.266, *p* < 0.001) and behavioral intention to use (β = 0.340, *f*^2^ = 0.125, *p* < 0.001), supporting H4 and H5. The results found that technology readiness was positively and significantly related to perceived usefulness (β = 0.209, *f*^2^ = 0.101, *p* < 0.001) and perceived ease of use (β = 0.613, *f*^2^ = 0.347, *p* < 0.001), supporting H6 and H7. Research results showed that interactivity was positively and significantly moderating relationship between perceived ease of use and attitude toward using (β = 0.143, *f*^2^ = 0.042, *p* < 0.05), supporting H8; similarly, interactivity was also positively and significantly moderating relationship between perceived usefulness and attitude toward using (β = 0.129, *f*^2^ = 0.031, *p* < 0.05), supporting H9.

**TABLE 2:** Results of the hypotheses testing.

Paths	Coefficients	*t*-value	Results
H1: Attitude toward using → Behavioral intention to use	0.601	9.924	Confirmed
H2: Perceived ease of use → Perceived usefulness	0.728	14.452	Confirmed
H3: Perceived ease of use → Attitude toward using	0.309	3.506	Confirmed
H4: Perceived usefulness → Attitude toward using	0.507	5.680	Confirmed
H5: Perceived usefulness → Behavioral intention to use	0.340	6.111	Confirmed
H6: Technology readiness → Perceived usefulness	0.209	4.029	Confirmed
H7: Technology readiness → Perceived ease of use	0.613	9.181	Confirmed
H8: Interactivity*Perceived ease of use → Attitude toward using	0.143	2.353	Confirmed
H9: Interactivity*Perceived usefulness → Attitude toward using	0.129	2.186	Confirmed

## Conclusion

### Discussion

After identifying MMKs in convenience stores as the research subject, we discussed literature on consumer behavior theories at first. Then, we found out other factors that may affect consumer behaviors by reference to qualitative research on multimedia and multi-channel context (Shankar and Yadav, 2010; Hsiao et al., 2020; Brengman et al., 2021; Lao et al., 2021). Finally, we selected suitable theories to express these factors, and verified these factors in a quantitative method (Wallace and Sheetz, 2014; Kim et al., 2017; Min et al., 2019). Previous studies on MMKs have not fully investigated both the factors that influence consumers’ intention to use and the continued use of various functions of MMKs (Chang et al., 2016; Hsiao et al., 2020). Similar to previous studies (Oh et al., 2014; Kim and Chiu, 2019; Chiu and Cho, 2020; Chang and Chen, 2021), this study is based on TAM and combined with the role of technology readiness emphasized by Lin et al. (2007), and introduces the moderating variable of interactivity, as an extension to TRAM, to verify consumers’ attitude toward using and behavioral intention to use.

First of all, this study assumes that technology readiness has a positive effect on perceived usefulness and perceived ease of use in TAM. Research findings show that the hypothesis is supported, which means that technology readiness indeed has a significant and positive impact on the two factors, and is an important antecedent in TRAM. This result is consistent with the conclusion of previous studies (Walczuch et al., 2007; Meng et al., 2009; Gupta and Garg, 2015; Lin and Kim, 2016) that technology readiness is able to effectively predict the adoption of technology media and improve user experience (Oh et al., 2014; Parasuraman and Colby, 2015; Chen and Lin, 2018; Kim and Chiu, 2019; Chiu and Cho, 2020). This means that the adoption rate will be enhanced (Chiu and Cho, 2020) if MMK users believe that the technology is identical and harmonious with their behaviors, habits, values and demands (Liébana-Cabanillas et al., 2015; Wang et al., 2020). However, the results of this study are found contrary to those of Humbani and Wiese (2019). They believed that it is the differences in research situations that lead to different research results in the context of developing countries by American/Western theories (Duh, 2015). This statement supports the research results of this study. Even if in the oriental society, the differences in consumer usage behavior among developed countries will reduce and thus produce similar level of technology adoption.

Second, this study assumes that there is a positive effect among perceived ease of use, perceived usefulness and attitude toward using in the original TAM. Research findings show that perceived ease of use has a positive and significant impact on perceived usefulness and attitude toward using, and that perceived usefulness has also the same impact on attitude toward using. The results are similar to those of Liébana-Cabanillas et al. (2015), and Humbani and Wiese (2019). They believed that in TRAM, perceived ease of use can effectively enhance consumer satisfaction with new technologies and indirectly affect consumers’ attitude toward the continued use of new technologies, and particularly, perceived usefulness is an important moderator (Rauniar et al., 2014; Wallace and Sheetz, 2014; Rafique et al., 2020; Wang et al., 2020). This implies that consumers will believe MMKs are useful if fewer resources or little time are/is required to learn how to use them, and the attitude toward using them continuously will also be affected.

In terms of the correlation of perceived usefulness and attitude toward using with behavioral intention to use, the findings show that perceived usefulness and attitude toward using have a positive and significant impact on behavioral intention to use. This is identical with the argument of Humbani and Wiese (2019). They believed that consumers will have a satisfied attitude toward new technologies, thus enhancing the force for continued use if they recognize the improvement of their life and work quality brought by new technologies. At the same time, again, this study also verifies the research findings of Rafique et al. (2020) that consumer satisfaction with the use of new technologies will directly determine the degree of behavioral intention to use.

Finally, yet importantly, this study combines interactivity with TRAM to discuss the moderating effect of perceived ease of use and perceived usefulness. The results show that interactivity will positively and significantly moderate the relationships among perceived ease of use, perceived usefulness and attitude toward using. Interactivity is introduced in this study to express consumers’ view on the use experience of web services (Zhao and Lu, 2012). According to the WAM proposed by Castañeda et al. (2007), consumers’ web use experience has a moderating effect on the user behavior of web services. This study reaches the same conclusion with Castañeda et al. (2007) in terms of this effect. In other words, MMKs are also a kind of web-based interactive technology (Shankar and Yadav, 2010; Barreda et al., 2016; Kim and Lee, 2019), and its self-help service is highly similar to other web services, making a lower cognitive difference in terms of usage among consumers.

### Managerial Implications

Based on the above research findings, several managerial implications are put forward as follows. First, this study proves that perceived ease of use can obviously enhance consumers’ perceived usefulness and attitude toward using MMKs. This means that reducing the required time to learn how to use MMKs is an important direction. This study suggests that managers of convenience stores should develop APPs connected with MMKs to shorten the operation time on MMKs. In this way, consumers can operate on the APP in advance, and can obtain the bill of payment only by entering a service code or scanning the QR code on an MMK. The free operation at any time anywhere is integrated with the sense of payment security brought by the closed system of MMKs, which is certain to bring convenient and fast operating experience for consumers.

Second, this study finds that interactivity has a significant and positive moderating effect on perceived ease of use and perceived usefulness. This implies that the user-friendly interface of new technologies contributes to consumers’ adoption of them. However, it would take a quite long time to develop an APP that integrates all information of more than 50 types of services, which may involve a variety of industries, provided by MMKs in convenience stores. This study suggests to start from helpful and frequently used items according to the 80/20 rule. We believe that this will realize great improvements in usage of MMKs in convenience stores in a short period of time.

Furthermore, as shown in the statistical results about technology readiness in this study, optimism contributes the most to consumers’ willingness to use mobile phones for consumption. Thus, this study also suggests that a discount section should be designed on the APP in order to improve the positive impression and raise the usage rate of the APP.

### Limitations and Future Research Directions

This study is the primary step toward understanding the behavioral intention to use MMKs at 24-h chain convenient stores, and the research is expected to extend usage in Taiwan, which is a developed economy. In this study, TRAM is explored, and significant insights are proposed through interactivity. Nevertheless, there are still some limitations in the study. First, this study adopts cross-sectional survey and investigates the consumers in Taiwan. Although it has few geographic limitations, there are still certain restrictions on generalizing the findings in other contexts. As discussed by Humbani and Wiese (2019), geographic location and economic development still have a significant impact on the application of TRAM. Thus, this survey can be extended to other eastern developing countries across various cultures on a comparative ground. In addition, due to the use of sampling, the results are prevented to be generalized and future studies may replicate the model in representative data. Second, using methods suggested by predecessor scholars, we analyzed the four potential variables in technology readiness at the first-level construct. But some scholars argued that they should be discussed at positive and negative constructs separately. Therefore, we suggest that researchers in the future should discuss the impact of technology readiness on TAM from the positive and negative constructs to offer more diversified insights. Third, this study discusses the moderating role of interactivity in the relationship marketing, but TAM is a theoretical model of high pluralism and inclusiveness, so we suggest that researchers in the future further discuss the establishment and development of the model by reference to other different theories in order to enrich it.

## Data Availability Statement

The raw data supporting the conclusions of this article will be made available by the authors, without undue reservation.

## Ethics Statement

The studies involving human participants were reviewed and approved by the Academic Committee of School of Economics and Management of Foshan University. The patients/participants provided their written informed consent to participate in this study.

## Author Contributions

Both authors listed have made a substantial, direct, and intellectual contribution to the work, and approved it for publication.

## Conflict of Interest

The authors declare that the research was conducted in the absence of any commercial or financial relationships that could be construed as a potential conflict of interest.

## Publisher’s Note

All claims expressed in this article are solely those of the authors and do not necessarily represent those of their affiliated organizations, or those of the publisher, the editors and the reviewers. Any product that may be evaluated in this article, or claim that may be made by its manufacturer, is not guaranteed or endorsed by the publisher.
